# Serological evidence of SARS-CoV-2 infection in dromedary camels and domestic bovids in Oman

**DOI:** 10.1080/22221751.2023.2220577

**Published:** 2023-06-12

**Authors:** Ihab El Masry, Salim Al Makhladi, Mohsin Al Abdwany, Afrah Al Subhi, Hatim Eltahir, Samuel Cheng, Malik Peiris, Emma Gardner, Sophie Von Dobschuetz, Baba Soumare, Madhur Dhingra, Keith Sumption, Markos Tibbo

**Affiliations:** a Food and Agriculture Organization of the United Nations, Rome, Italy; b Animal Health Research Center, Ministry of Agriculture, Fisheries and Water Resources, Muscat, Oman; c School of Public Health, Li Ka Shing Faculty of Medicine, The University of Hong Kong, Hong Kong SAR, People’s Republic of China; d Food and Agriculture Organization of the United Nations, Subregional Office for the Gulf Cooperation Council States and Yemen, Abu Dhabi, UAE

**Keywords:** SARS-CoV-2, Livestock, Bovids, Dromedary camel, COVID-19

## Abstract

SARS-CoV-2 has demonstrated the ability to infect a wide range of animal species. Here, we investigated SARS-CoV-2 infection in livestock species in Oman and provided serological evidence of SARS-CoV-2 infection in cattle, sheep, goats, and dromedary camel using the surrogate virus neutralization and plaque reduction neutralization tests. To better understand the extent of SARS-CoV-2 infection in animals and associated risks, “One Health” epidemiological investigations targeting animals exposed to COVID-19 human cases should be implemented with integrated data analysis of the epidemiologically linked human and animal cases.

Severe acute respiratory syndrome coronavirus 2 (SARS-CoV-2), the causative agent of the ongoing Coronavirus Disease 2019 (COVID-19) pandemic in humans, has demonstrated the ability to infect a wide range of animal species. As of December 2022, more than 39 countries, predominantly in Europe and the Americas, have reported SARS-CoV-2 infections in animals [1]. Natural infections have been confirmed in 30 different animal species [2]. Positive findings in animals have raised concerns about the possible role they play in amplifying and spreading the virus and establishing reservoirs [3]. Studies that investigate transmission within animal species would elucidate the transmission dynamics of SARS-CoV-2 and the potential role different animal species may play in the ecology of the virus and the risk to humans and animals.

This study aimed to investigate evidence of SARS-CoV-2 infection in livestock species in Oman where more than 390,000 confirmed human cases of COVID-19 between January 2020 and January 2023 were reported [4]. The predominant type of livestock species are goats (2,433,000), sheep (642,000), cattle (421,000), and dromedary camels (285,000) [5].

By end of March 2022, the number of COVID-19 human cases in Oman was at low level below 500 cases per week and continued as such until end of June 2022 [4]. Between 8 and 30 June 2022, 617 serum samples were collected randomly from 54 cattle, 176 sheep, 207 goats, and 180 dromedary camels covering 31 villages in 11 districts (wilayat) of Oman. The samples were collected by scientists of the Animal Health Research Center. Three to seven animals were sampled from each herd. In some exceptional cases, however, up to 10 animals were sampled per herd when the herd size was greater than 40 animals. No samples were collected for the detection of the virus RNA which is out of the scope of this study.

The collected sera were stored in −20°C and transferred to the virology laboratory at the University of Hong Kong where testing was conducted in August 2022. The sera were initially screened for the presence of SARS-CoV-2 antibodies using the surrogate virus neutralization test (sVNT) (GenScript cPass™ SARS-CoV-2 Neutralization Antibody Detection Kit, Genscript, The Netherlands), the test was performed according to the manufacturer’s instructions. Positive sera (Cutoff 30%) were further tested by plaque reduction neutralization test (PRNT) against wild type (WT) virus as described elsewhere [6]. Serial dilution of sera from a dilution of 1:10 to 1:320 were tested. The highest serum dilutions resulting in ≥50% reduction of plaque numbers (PRNT_50_) and 90% (PRNT_90_) were recorded. A PRNT_50_ antibody titre ≥1:10 was considered positive.

Six animals (3 goats (1.45%), 1 sheep (0.57%), 1 cattle (1.85%), and 1 camel (0.56%)) tested positive in both sVNT and PRNT_50_ assays, and another 2 camels tested positive by sVNT and negative by PRNT_90_ and PRNT_50_ so their results considered as seronegative (Table 1). These results demonstrate likely serological evidence of natural SARS-CoV-2 infection in these species. The low numbers of seropositive animals suggest that the exposed animals had likely acquired the infection from contact with COVID-19 human cases, however, animal-to-animal transmission cannot be ruled out.

**Table 1.** Demographic data of the animals that tested positive for SARS-CoV-2 antibodies by sVNT and PRNT assays.

Species	Wilayat (districts)	Village	Sex	Age (Months)	sVNT % inhibition	PRNT_90_ titre	PRNT_50_ titre	PRNT result
Camel1	Barka	Al Wahra	Female	12	44.8	<1:10	<1:10	Negative
Camel2	Al Suwaiq	Dhian	Female	6	37.6	<1:10	<1:10	Negative
Camel3	Al Mudhaibi	Al Washehi	Female	7	89.3	1:40	1:160	Positive
sheep	Al Masenaa	Bu Asim	Female	3	82.9	1:10	1:40	Positive
Goat1	Barka	A’Noaman	Female	11.5	44.0	<1:10	1:10	Positive
Goat2	Al Masenaa	Bu Asim	Female	5	39.6	<1:10	1:20	Positive
Goat3	Al Masenaa	Bu Asim	Female	4	46.6	<1:10	1:10	Positive
Cattle	Barka	Al Hafri	Male	2	76.0	1:10	1:40	Positive

The age of the seropositive animals ranged from two to twelve months old, suggesting that they may have been exposed to one or more of the SARS-CoV-2 variants circulating between June 2021 and June 2022. This period can be divided into a pre-Omicron period (June-November 2021), in which 318 (88%) of sequences submitted to GISAID are identified as Delta variant of concern (VOC), and an Omicron period (December 2021-June 2022) in which 54 (50%) sequences were identified as Omicron BA.1 and sublineages, 32 (30%) as BA.2 and sublineages, and 2 (2%) as BA.5 sublineages [7]. The remaining proportion of sequences submitted during the Omicron period are most likely residual Delta sequences during the delta-omicron transition in December 2021.

Previous in-silico studies have suggested a potential susceptibility of cattle, sheep, goat and camels to the WT virus [8]. Although cattle experimentally infected with SARS-CoV-2 did not seroconvert at 14 days post infection (DPI) using PRNT_90_ [9], SARS-CoV-2 antibodies have been detected in cattle through field surveys in Germany using immunofluorescence or a surrogate neutralization assay [10] and in Italy using electrochemiluminescence immunoassay and Microneutralisation Test [11]. Similarly, none of the sheep, goats and camelids (alpaca) experimentally infected with SARS-CoV-2 had seroconverted at 14 DPI [9]. It is known that the host range may depend on the virus variant, with mice not being susceptible to infection with the original WT virus but mice were susceptible to the Alpha variant [12]. Thus, lack of evidence of experimental infection with the wild-type variant may not preclude infection with other variants. To our knowledge, our study presents the first field serological survey to report detection of SARS-CoV-2 antibodies in sheep, goats, and dromedary camels. Moreover, the sVNT and PRNT antibody titres observed in three species (cattle, sheep, and camel 3) in three different geographical areas of Oman are high enough to suggest these are likely not cross reacting with other coronaviruses, although this cannot be ruled out.

One of the limitations of this study is that the sampling strategy was not statistically representative of the targeted animal population. Furthermore, there is not enough data on the VOC circulating in Oman during the relevant time period, especially Omicron sub-variants. Several studies have reported reduced cross neutralization not only between Omicron and other VOCs but also between some of the Omicron sub-variants [13]. Therefore, the results cannot be extrapolated to reflect the extent of SARS-CoV-2 infection in the targeted species in Oman, given the use of WT virus in the employed PRNT assays. Furthermore, we only tested for antibody to the original virus and not to more recent virus variants and waning antibody titres may also contribute to false negative results. Thus, our estimates of SARS-CoV-2 infection in sampled animals may be less than the actual value. Exposure history of the sampled herds to COVID-19 patients is unknown, therefore the serological evidence of animal infection being a result of zoonotic transmission is an assumption.

This study provided evidence of SARS-CoV-2 infection in cattle, sheep, goats, and dromedary camels under field conditions. The role different animal species may play in the evolution or sustaining the circulation of SARS-CoV-2 and the potential consequences on animal and human health is an important area of research. To better understand the extent of SARS-CoV-2 infection in different animal species and the associated risks, “One Health” epidemiological investigations of SARS-CoV-2 transmission to animals exposed to COVID-19 human cases should be implemented according to international standards with integrated data analysis of the epidemiologically linked human and animal cases [3], and attention to different species of livestock, companion, and wild animals in close proximity to humans.

## Supplementary Material

Supplemental Material
