# Supplementary material for: Serological evidence of SARS-CoV-2 infection in dromedary camels and domestic bovids in Oman
Source: Emerg Microbes Infect. 2023 Jun 12;12(1):2220577. doi: 10.1080/22221751.2023.2220577 (PMC10262771; doi:10.1080/22221751.2023.2220577)
Supplement: Supplemental Material [file TEMI_A_2220577_SM7636.docx]

Serological evidence of SARS-CoV-2 infection in dromedary camels and domestic bovids in Oman

Ihab El Masry^1^
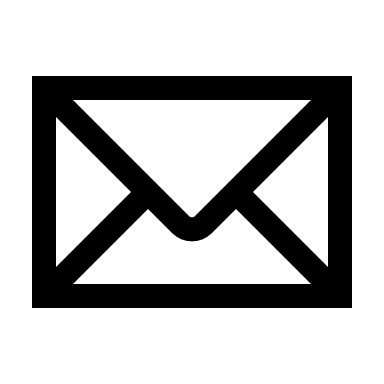
, Salim Al Makhladi^2^, Mohsin Al Abdwany^2^, Afrah Al Subhi^2^, Hatim Eltahir^2^, Samuel Cheng^3^, Malik Peiris^3^, Emma Gardner^1^, Sophie Von Dobschuetz^1^, Baba Soumare^1^, Madhur Dhingra^1^, Keith Sumption^1^, Markos Tibbo^4^

Supplementary figure. Map of the sampling areas and the areas of seropositive animals


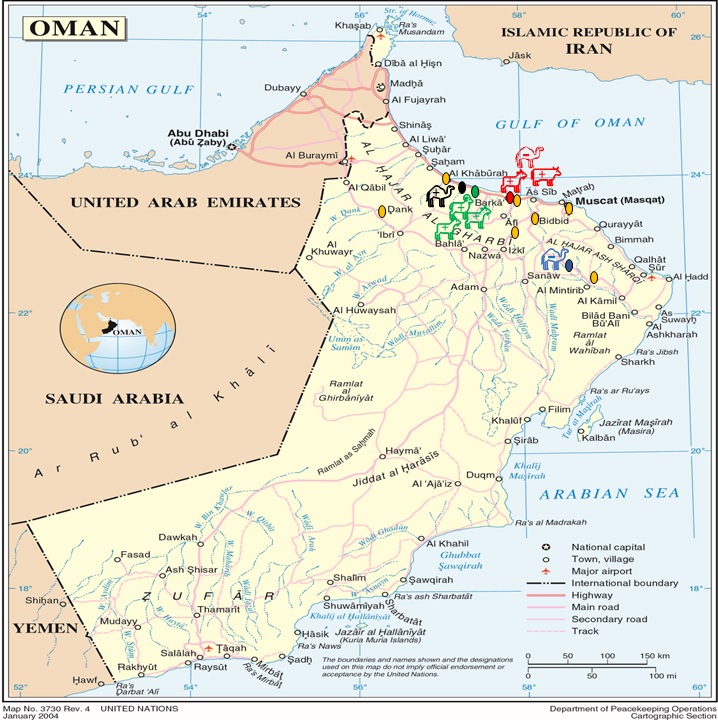


- Yellow circles: sampling areas where none of the sampled animals tested positive.
- Non-Yellow circles: sampling areas where at least one animal tested positive by either sVNT or PRNT.
- +: animal tested positive for both sVNT and PRNT.
- -: animal tested positive for sVNT but negative for PRNT.
- Black camel: sampled in Al Mudhaibi Wilayat
- Green sheep and goats: sampled in Al Masenaa Wilayat
- Red camel, goat and cattle: sampled in Barka Wilayat
- Blue camel: sampled in Al Suwaiq Wilayat

Source: United Nations Geospatial. 2014. Map of Oman. United Nations. Cited 14 April 2023. <https://www.un.org/geospatial/content/oman>.

Disclaimer: The boundaries and names shown and the designations used on these map(s) do not imply the expression of any opinion whatsoever on the part of FAO concerning the legal status of any country, territory, city or area or of its authorities, or concerning the delimitation of its frontiers and boundaries.
